# Zebrafish (*Danio rerio*) as a Model for the Study of Developmental and Cardiovascular Toxicity of Electronic Cigarettes

**DOI:** 10.3390/ijms25010194

**Published:** 2023-12-22

**Authors:** Eman Hussen, Nada Aakel, Abdullah A. Shaito, Maha Al-Asmakh, Haissam Abou-Saleh, Zain Z. Zakaria

**Affiliations:** 1 Biological Science Program, Department of Biological and Environmental Sciences, College of Arts and Sciences, Qatar University, Doha P.O. Box 2713, Qatar; eh1800888@qu.edu.qa; 2 Biomedical Sciences Department, College of Health Sciences, Qatar University, Doha P.O. Box 2713, Qatar; na1709483@qu.edu.qa (N.A.); maha.alasmakh@qu.edu.qa (M.A.-A.); hasaleh@qu.edu.qa (H.A.-S.); 3 Biomedical Research Center, Qatar University, Doha P.O. Box 2713, Qatar; abdshaito@qu.edu.qa; 4 Medical and Health Sciences Office, QU Health, Qatar University, Doha P.O. Box 2713, Qatar

**Keywords:** electronic cigarettes, tobacco, cardiotoxicity, Zebrafish

## Abstract

The increasing popularity of electronic cigarettes (e-cigarettes) as an alternative to conventional tobacco products has raised concerns regarding their potential adverse effects. The cardiovascular system undergoes intricate processes forming the heart and blood vessels during fetal development. However, the precise impact of e-cigarette smoke and aerosols on these delicate developmental processes remains elusive. Previous studies have revealed changes in gene expression patterns, disruptions in cellular signaling pathways, and increased oxidative stress resulting from e-cigarette exposure. These findings indicate the potential for e-cigarettes to cause developmental and cardiovascular harm. This comprehensive review article discusses various aspects of electronic cigarette use, emphasizing the relevance of cardiovascular studies in Zebrafish for understanding the risks to human health. It also highlights novel experimental approaches and technologies while addressing their inherent challenges and limitations.

## 1. Introduction

Smoking involves the combustion of tobacco products and the inhalation or expulsion of resulting smoke. This practice dates back to around 5000–3000 BC in South America and Mesoamerica [1]. Cigarettes gained popularity due to their convenience and affordability; however, studies have increasingly linked cigarette smoking to various health problems [2]. Short-term smoking effects, such as coughing, wheezing, and antioxidant depletion, can become evident immediately or shortly after smoking [3,4]. The long-term health effects, including cardiovascular disease, chronic lung disease, diabetes, and cancer, are significant causes of death among smokers [5]. Smoking contributes to the development of cardiovascular diseases such as ischemic heart disease and stroke, the primary causes of mortality worldwide [5,6]. Smoking’s risks during pregnancy are well-established, raising the risks of neurodevelopmental issues, depression, intrauterine growth problems, hyperactivity, and cognitive impairments [7].

Historically, cigarettes were linked to the lung cancer epidemic in the 1940s and 1950s, with evidence from epidemiology, animal studies, cellular pathology, and chemical analysis [8]. Over time, cancer-causing chemicals in cigarette smoke, including polycyclic aromatic hydrocarbons and benzpyrene, were discovered [8,9]. It is widely accepted that traditional cigarette smoke adversely affects health, contributing to lung cancer and respiratory problems like chronic obstructive pulmonary disease (COPD). Despite declining consumption rates, smoking-related lung cancer deaths are projected to increase by 2020–2030 [8,10].

In response to the adverse health effects of traditional cigarettes, people have turned to alternative tobacco products, especially in high-risk situations such as pregnancy. The e-cigarette, designed as a safer alternative to regular tobacco cigarettes, administers nicotine without tobacco combustion [11]. Encouraged by campaigns endorsing their safety, the sales of e-cigarettes have grown exponentially [12]. However, evidence about the safety and impact of electronic cigarettes on human health is limited, and their long-term effects are not fully understood. A study has reported that heating processes in e-cigarettes may generate new compounds with potential toxicity to human health [13]. Despite claims that e-cigarettes contain fewer toxic compounds than regular cigarettes, there is limited scientific evidence regarding the adverse outcomes of e-cigarette smoking, even at low levels, especially during pregnancy [11]. Recent studies show that exposure to e-cigarettes has adverse effects on various age groups, including fetuses and newborns [14].

The concept of electronic cigarettes was developed by pharmacist Hon Lik in 2004 [15], with the aim of obtaining nicotine with fewer toxins. Marketing has labeled e-cigarettes as a “healthier alternative” to traditional cigarettes [16]. Following the introduction of electronic cigarettes to the market, heated tobacco products (HTPs) were introduced, including Glo manufactured by British American Tobacco (BAT) and the Tobacco Heating System (THS) or IQOS by Philip Morris International (PMI). However, there is still a debate about the safety of these devices, and their overall impact on public health is still unclear. Some research suggests that electronic cigarettes might be effective in reducing cigarette consumption. However, electronic cigarettes’ long-term carcinogenic and lung function effects remain undetermined [17].

The primary objective of this review article is to provide a comprehensive overview of electronic cigarette (e-cigarette) use and the current state of research on e-cigarettes. It also delves into the use of Zebrafish as a model for studying the developmental toxicity of e-cigarettes. With the increasing popularity of e-cigarettes and their potential impact on public health, this review aims to achieve several key objectives: synthesizing the existing literature, exploring aspects of e-cigarette consumption, including their chemical composition and implications, and emphasizing the significance of Zebrafish cardiovascular and developmental studies in understanding potential human health consequences. The article further investigates emerging experimental methodologies and technologies within this field, addressing related challenges and limitations. Ultimately, this review endeavors to equip readers with valuable insights into the intricate landscape of e-cigarette research and its far-reaching implications for public health and policy decisions.

## 2. E-Cigarettes in Smoking Cessation and Harm Reduction

Electronic cigarettes, commonly known as vapes, have gained popularity as battery-operated devices that deliver nicotine and other substances to users through an aerosol [18]. These devices heat a liquid solution containing nicotine, flavors, and additives, generating an inhalable aerosol that simulates smoking without combustion [19,20,21]. Initially marketed as a harm-reduction tool for smokers aiming to quit or reduce tobacco use, e-cigarettes were perceived as a less harmful alternative due to the absence of tobacco combustion [22]. Consequently, they were seen as both a smoking cessation aid and a means to mitigate the health risks associated with tobacco smoking [23,24]. Nevertheless, their widespread availability and marketing have led to their adoption by non-smokers, sparking concerns about their potential as a gateway to tobacco use among young individuals [22,25].

**Figure 1:**
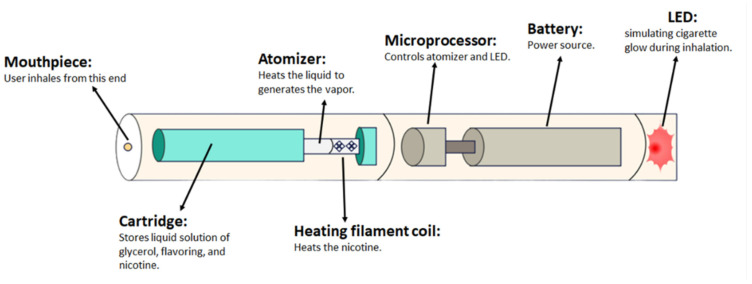
Electronic cigarette device.

The typical e-cigarette device comprises three primary components: a plastic tube, an electronic heating element, and a liquid nicotine cartridge, all powered by a lithium battery, as illustrated in Figure 1. Users activate the device by pressing a button, which releases a puff of vaporized nicotine [15]. Fueled by the lithium battery, the heating element vaporizes the liquid within the cartridge, which is then inhaled by the user, and commonly referred to as a ‘vaper’.

E-cigarette liquids encompass approximately 60 to 70 compounds, including nicotine, propylene glycol, glycerol, various flavors, and impurities like cotinine and nornicotine [26,27,28]. In Table 1, shows the key compounds of E-cigarettes and their adverse effects to humans. Nicotine, a highly addictive stimulant, can adversely affect brain development and cardiovascular systems [29]. Extensive research highlights the detrimental impact of nicotine exposure on various body systems, particularly during critical developmental phases, resulting in disruptions to brain maturation and cardiovascular abnormalities [30,31,32]. Propylene glycol, categorized as safe for ingestion, can lead to respiratory issues and asthma when inhaled, posing risks to individuals with impaired kidney or liver function [26]. E-liquids often contain toxic carbonyl compounds like acetaldehydes and formaldehyde, which are associated with carcinogenic effects and other health problems [26]. Over 7000 e-liquid flavors lack comprehensive research on their heated inhalation effects, and compounds like diacetyl and acetyl propionyl are linked to risks such as ‘Popcorn lung’ disease, while certain flavoring compounds induce oxidative stress and cytotoxic effects [33,34]. Although recognized as safe for ingestion by the FDA, the safety of inhaling heated compounds remains unclear [35]. Additionally, e-cigarettes carry a high risk of toxic impurities, including heavy metals and tobacco-specific nitrosamines [36]. Carbonyl compounds in e-cigarette vapor can cause mouth and throat irritation, while exposure to formaldehyde, acetaldehyde, and acrolein in rats demonstrates severe toxic and irritating effects [37].

Factors such as perceived harm reduction, appealing flavors, and easy access through social media and online marketing have contributed to the popularity of e-cigarettes [38]. While some consider them a valuable tool for smoking cessation, particularly with claims of reduced harmful chemicals, their effectiveness remains debatable [23,39].

When studying the components of e-liquid, a wide variety of flavors are utilized, serving as a significant factor in promoting initiation and higher usage frequency among young users [40]. Various flavors are available in marketing, including tobacco, menthol/mint, fruit, sweet/dessert, and drink flavors [41]. An international survey of adult former smokers indicated that flavor diversity played a ‘significant’ role in their attempts to quit smoking, reduce enjoyment, and ease the transition from smoking to using e-cigarettes [40]. Certain flavors, like butter-flavoring diacetyl, can trigger inflammation and bronchiole scarring [42], while others, such as cinnamaldehyde and vanillin, promote oxidative stress and cytokine release, hindering innate immune responses [42,43].

Several studies have indicated that e-cigarettes contain significantly lower concentrations of carcinogens and toxins than regular cigarette smoke, potentially making them practical for smoking cessation [37,44]. However, uncertainty prevails regarding whether the low levels of toxins in e-cigarettes fall below the threshold for human health risk [45,46].

A study by Barbeau et al. reported that e-cigarettes help some tobacco smokers transition to a less harmful replacement tool, thereby maintaining cigarette abstinence [47]. In a randomized controlled trial conducted in New Zealand with 657 smokers, the efficacy of e-cigarettes was compared to nicotine patches or placebo e-cigarettes (non-nicotine). Verified abstinence rates after 6 months were 7.3% for nicotine e-cigarettes, 5.8% for patches, and 4.1% for placebo e-cigarettes. E-cigarettes exhibited moderate effectiveness similar to nicotine patches, but their precise role in tobacco control remains uncertain, necessitating further research to clarify benefits and risks [47,48]. In contrast, a German study explored the use of e-cigarettes as an alternative approach in a smoking cessation study. It found that while 12.6% of participants used e-cigarettes during or after the intervention, those who initially smoked more and were more addicted to cigarettes found it less successful in achieving tobacco abstinence than nicotine replacement therapy or no additional cessation aids. Integrating e-cigarettes into abstinence-oriented smoking cessation groups might be counterproductive due to a lack of clear usage guidelines and potential distractions from quitting motivation [49].

**Table 1:** Key compounds of E-cigarettes and their adverse effects.

Compound	Adverse Effects and References
Nicotine	Brain maturation disruptions and cardiovascular abnormalities [30,31,32]
Propylene glycol	Respiratory issues and asthma [26]
Carbonyl compounds (acetaldehydes and formaldehyde)	Carcinogenic effects, mouth irritation and throat irritation [26,37]
Diacetyl and acetyl propionyl	‘Popcorn lung’ disease [33,34]
Butter-flavoring diacetyl	Inflammation and bronchiole scarring [42]
cinnamaldehyde and vanillin-flavoring	Promote oxidative stress and cytokine release [42,43]

## 3. Zebrafish as a Versatile Model for Health Research

As the use of e-cigarettes continues to rise, exploring associated health risks, especially among vulnerable populations, becomes increasingly critical. Traditional mammalian toxicity studies, aside from being costly and ethically challenging [50,51], are now finding alternatives in Zebrafish, primarily due to their genetic and physiological similarities to humans [52,53]. Zebrafish offer distinct advantages, including their external embryonic development, which enables efficient monitoring, quicker experiments, and real-time visualization. Their high reproductive rates ensure statistically significant studies, and the transparency of zebrafish embryos facilitates cost-effective drug testing and safety assessments, expediting drug development while minimizing risks [54,55]. Furthermore, their genetic manipulability aids in gaining mechanistic insights, and their transparency allows for internal organ visualization during toxicological research, reducing the reliance on mammalian models and addressing ethical concerns [53,56].

Zebrafish have proven valuable for evaluating the harm associated with tobacco, demonstrating sensitivity to both smoke and nicotine [57,58]. They reveal developmental abnormalities, reduced survival rates, and adverse behavioral effects [59,60], providing insights into nicotine-related diseases, craniofacial defects, and behavioral impacts. This underscores their potential in studying the health risks associated with e-cigarette usage [61,62].

## 4. Zebrafish as an Insightful Model for Cardiovascular Research

**Figure 2:**
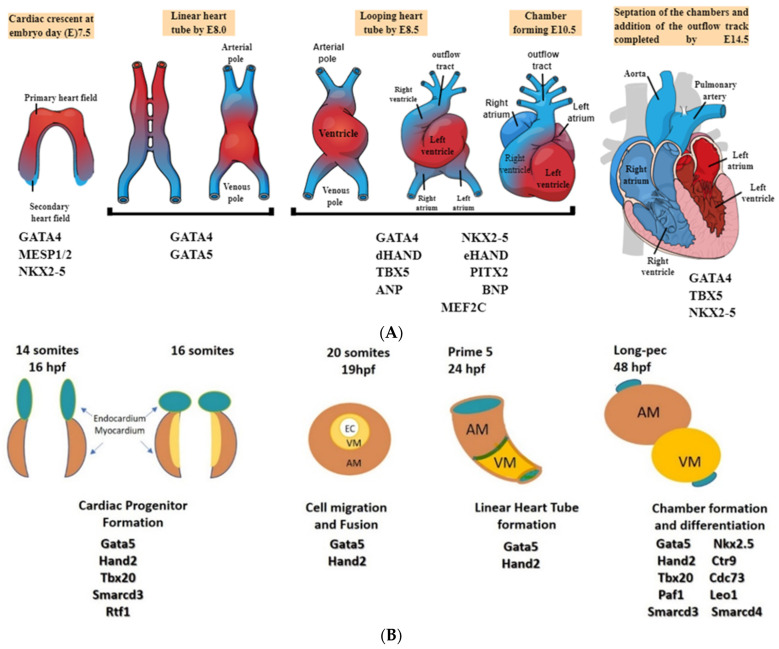
Comparison of cardiovascular development in humans and zebrafish: Exploring signaling pathways governing cardiomyocyte induction. (**A**) Human heart development. (**B**) Zebrafish heart development [52].

Zebrafish (*Danio rerio*) have emerged as a significant model organism for investigating cardiovascular development, primarily due to the striking resemblance between their cardiovascular systems and humans, as shown in Figure 2 [63]. Zebrafish cardiovascular development encompasses several key stages, each holding immense relevance for advancing cardiovascular research; please refer to Table 2. Leveraging Zebrafish as a model organism has yielded invaluable insights into heart development, cardiovascular diseases, and potential therapeutic strategies [64,65,66]. Zebrafish also provide critical insights into cardiovascular diseases and regenerative medicine concerning heart repair [67,68,69].

The cardiovascular development of zebrafish is pivotal in advancing our understanding of various critical aspects of human health. The shared conservation of essential genes and signaling pathways between zebrafish and humans provides valuable insights into the intricate processes of heart development and the mechanisms behind cardiovascular diseases; please refer to Table 2 [70]. Zebrafish are invaluable models for replicating human cardiovascular conditions, allowing an in-depth exploration of disease mechanisms and processes [71]. Additionally, the zebrafish’s remarkable ability to regenerate heart tissue, a feature absent in humans, offers significant insights into potential regenerative strategies for repairing cardiac tissue [72,73,74,75]. Researchers can uncover the functional consequences of disease-associated genes through genetic manipulation of the zebrafish, contributing to our understanding of human cardiac disorders [69,75].

Moreover, zebrafish models open doors to personalized medicine, enabling the testing of patient-specific genetic variants and customizing treatment approaches [76,77,78]. In Table 3, we highlight the importance of zebrafish cardiovascular development as a powerful tool with extensive implications for enhancing human heart health, advancing diagnostics, developing therapies, and fostering innovation in drug development.

**Table 2:** Key stages of Zebrafish (*Danio rerio*) cardiovascular development and their relevance to cardiovascular research.

Stage	Description	Significance and References
Early cardiac morphogenesis	Bilateral cardiac progenitor cells fuse to form a linear heart tube within 24 h post-fertilization (hpf).	Crucial initial step in heart development. Similarities to human aid study [79].
Chamber formation and looping	Remodelling from 24 to 48 hpf forms distinct chambers, and looping results in a single-looped heart.	Key stage for atrium and ventricle differentiation [80].
Valve development	Atrioventricular and bulboventricular valves develop and mature around 48 hpf.	Critical for regulating blood flow and potential insights into valve defects [81].
Onset of blood circulation	Blood circulation starts at 48 hpf as the heart beats, delivering oxygen and nutrients.	Foundation of nutrient transport, tissue development [82,83].
Later development and heart maturation	Further maturation occurs between 72 to 96 hpf, resulting in a fully developed atrium and ventricle with functional valves.	Increasing heart rate, organized blood flow [84].
Adult heart structure	Adult zebrafish heart has two chambers; atrium and ventricle. Atrium serves as a common chamber.	Unique structure compared to humans; fundamental processes conserved [85,86].
Transparency and genetic manipulation	Transparent embryos allow live imaging and genetic manipulation for studying gene roles and signaling pathways.	Powerful tool for cardiovascular research. Insights into gene functions [87,88,89].
Relevance to human cardiovascular research	Similarities aid understanding congenital heart defects, potential therapies, regenerative strategies.	Translational implications for human cardiovascular diseases and repair [63,86,90].

## 5. Exploring E-Cigarette Effects on Zebrafish: Innovative Exposure Methods for Cardiovascular and Developmental Assessments

**Figure 3:**
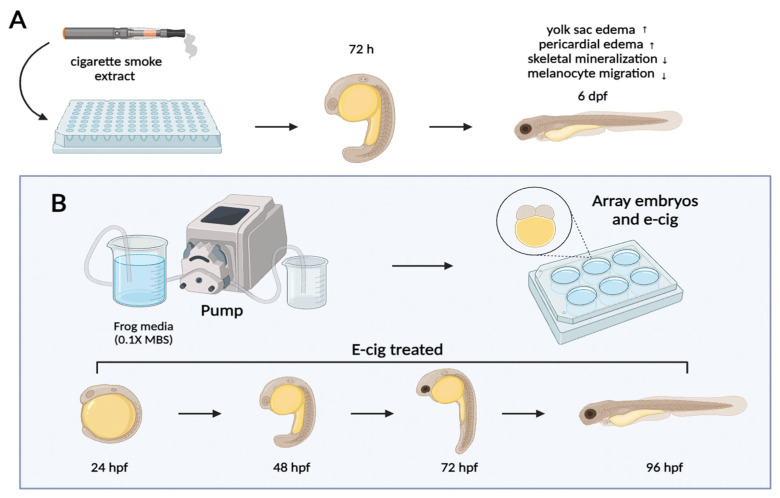
Methods used to expose zebrafish to e-cigarette aerosols or constituents. (**A**) The utilization of an e-cigarette exposure paradigm for both qualitative and quantitative study. The embryos are subjected to electronic cigarette (e-cig) exposure starting from the 2-cell stage at 1.5 h post-fertilization (hpf) and continuing until 72 hpf. (**B**) The experimental setup for embryonic specimens; The embryos are arranged in 6-well culture dishes, with a dilution ratio of 1:100 of e-cig. The analysis is conducted utilizing developmental phases as the basis for investigation in zebrafish.

Research investigating the impact of e-cigarette aerosols and their constituents on zebrafish involves carefully designed exposure experiments that aim to replicate real-world scenarios (Figure 3). These investigations aim to uncover potential consequences for zebrafish cardiovascular development and overall well-being. Multiple methodologies are employed to expose zebrafish to e-cigarette aerosols or their components, including:

1. Whole-body exposure: Researchers utilize a custom-built exposure chamber to expose adult zebrafish to e-cigarette aerosols in a whole-body setup. This study assesses cardiovascular parameters and examines gene expression effects [91].
2. Waterborne exposure: Zebrafish embryos are exposed to diluted e-cigarette e-liquids in their surrounding water. This approach explores the impact on embryonic development and behavior [92,93,94].

These exposure techniques allow researchers to monitor Zebrafish health and development aspects. Key parameters such as heart rate, cardiac morphology, blood vessel development, and relevant physiological indicators are commonly evaluated. The insights gained from these exposure methods contribute to our understanding of potential cardiovascular risks associated with e-cigarette use. Furthermore, these studies provide valuable insights into the broader implications of e-cigarettes on both human health and the environment.

## 6. Effects of E-Cigarette Exposure during Pregnancy and on Newborns

Using e-cigarettes during pregnancy raises concerns about potential fetal and maternal health risks. It is important to note that the safety of e-cigarette use during pregnancy is not well-established due to limited scientific evidence [95,96]. Some pregnant women turn to e-cigarettes as a harm-reduction strategy, believing they are less harmful than traditional cigarettes, but this perception lacks substantial support [97].

### 6.1. Fetal Growth and Structural Abnormalities

Research suggests that exposure to e-cigarettes during pregnancy may negatively affect placental function and result in fetal structural abnormalities. Several studies have indicated that both e-cigarette and traditional cigarette use are associated with a higher risk of having newborns classified as “small for gestational age” (SGA), meaning their size is smaller than expected for their gestational age [98]. Furthermore, the effects of maternal e-cigarette use may extend beyond one generation, potentially impacting the offspring of daughters [99].

### 6.2. Respiratory and Cardiovascular Effects

E-cigarette use can adversely affect newborns’ and fetuses’ respiratory and cardiovascular systems. Nicotine, a common component of e-cigarettes, can cross the placental barrier, leading to high concentrations of nicotine in fetal serum and amniotic fluid, increasing the risk of mortality and morbidity for the fetus and newborn [100]. Even nicotine-free e-cigarettes have been shown to affect placental function, potentially leading to reduced angiogenesis and trophoblast impairment [95].

### 6.3. Neurobehavioral and Developmental Impacts

Prenatal e-cigarette exposure has been linked to various neurobehavioral disorders in offspring. Studies indicate that such exposure may increase the sensitivity to neonatal hypoxic-ischemic (H.I.) brain injury, a condition that can lead to cognitive deficits and other neurological issues [101]. Additionally, it has been associated with impaired motor coordination, altered stress-coping strategies, and reduced cognitive function in offspring [102].

### 6.4. Renal System Concerns

While the adverse effects of e-cigarettes on the urinary system remain less documented, some studies suggest potential harm. E-cigarette aerosols may introduce reactive aldehydes, like acrolein, to the renal system through blood circulation, potentially causing kidney injury [103]. Research conducted on pregnant mice exposed to e-cigarettes during pregnancy revealed an impact on kidney development in their offspring, including a reduction in parameters, glomeruli numbers, and increased oxidative stress [104].

Overall, the health hazards of e-cigarette use during pregnancy and on newborns are primarily associated with the cardiovascular and respiratory systems. While there is limited scientific evidence regarding the urinary system, potential fetal and maternal health risks cannot be disregarded. It is crucial for expectant mothers to consult with healthcare professionals and make informed decisions regarding e-cigarette use during pregnancy, considering the potential risks to both themselves and their unborn child.

## 7. Cellular and Molecular Mechanisms of E-Cigarette Toxicity in Zebrafish

While the complete understanding of e-cigarette toxicity in zebrafish is an ongoing process, recent studies have illuminated some of the intricate cellular and molecular mechanisms involved. These investigations have highlighted several key pathways, with oxidative stress and inflammation taking center stage. Exposure to e-cigarette aerosols, which contain reactive oxygen species (ROS) and harmful compounds, triggers oxidative stress within zebrafish tissues. This results in elevated oxidative stress markers and the activation of inflammatory pathways [105,106,107]. Additionally, e-cigarette exposure in zebrafish embryos has been associated with DNA damage and programmed cell death, known as apoptosis. Activating these apoptotic pathways may contribute to tissue damage and hinder the normal development of organs [57,108,109,110]. Moreover, disruptions in critical developmental signaling pathways, such as Wnt and Notch, have been observed due to e-cigarette exposure, leading to abnormal tissue and organ development [111,112,113,114,115,116].

Furthermore, changes in gene expression have been identified in zebrafish exposed to e-cigarette aerosols. These alterations in gene regulation may result in the dysregulation of genes essential for various cellular processes, thereby contributing to the observed toxic effects [57,114,117,118,119,120]. Lastly, e-cigarette exposure has been found to impair cellular functions and disrupt cell differentiation in Zebrafish embryos, potentially initiating cascading effects on organogenesis and tissue maturation [109,121]. For a summary of critical cellular and molecular mechanisms identified in zebrafish studies, please refer to Table 4.

It is essential to recognize that the specific pathways leading to cardiovascular damage in zebrafish due to e-cigarette exposure may vary depending on the specific e-cigarette formulations used and the duration and intensity of exposure. Furthermore, the translation of findings from zebrafish to human health requires further investigation, as there may be species-specific differences in how e-cigarette aerosols affect cardiovascular pathways. Integrating findings from zebrafish studies with human clinical research will provide a more comprehensive understanding of the potential risks associated with e-cigarette use on cardiovascular health.

**Table 4:** Critical cellular and molecular mechanisms identified in zebrafish studies.

Mechanism	Description
Oxidative stress and inflammation	E-cigarette aerosols induce oxidative stress and activate inflammatory pathways in zebrafish tissues [105,106,107].
DNA damage and apoptosis	E-cigarette exposure leads to DNA damage and programmed cell death (apoptosis) in zebrafish embryos [108,109,110].
Disrupted developmental signaling pathways	E-cigarette exposure disrupts critical developmental signaling pathways like Wnt and Notch, affecting organ development [111,112,113,114,115,116].
Gene expression changes	E-cigarette exposure alters gene expression profiles (bcl2, casp8, hsp70, Cbsa) in zebrafish, affecting various cellular processes [57,114,117,118,119,120].
Impaired cell function and differentiation	E-cigarette exposure impairs cellular functions (cardiovascular system, bone, vascular, and cartilage development) and differentiation, impacting tissue maturation and organogenesis [94,109,121].

## 8. Comparison of E-Cigarette Impacts: Zebrafish vs. Human Studies

The comparison between zebrafish studies and human research findings regarding the impact of e-cigarette exposure is constrained, as both fields of study are still evolving. Nevertheless, we can draw some key observations based on the available data:

### 8.1. Cardiovascular Effects

Both zebrafish and human studies have reported cardiovascular effects following e-cigarette exposure. Zebrafish studies have revealed alterations in heart rate, heart morphology, and impaired vascular development [114,115,122]. Similarly, human studies have indicated changes in heart function, vascular health, and endothelial function among e-cigarette users [123,124]. For instance, Palpant et al. (2015) used zebrafish to examine cardiac effects from exposure to nicotine, conventional cigarette smoke, or e-cigarette vapor during early development [114]. Animals exposed to tobacco and e-cigarette smoke exhibited significantly more severe heart defects than those exposed to nicotine alone. However, the impact of e-cigarette flavorings, individually or in combination, on zebrafish development remains unexplored [114]. The lack of precise knowledge regarding e-cigarette cartridge constituents and concentrations presents a challenge.

### 8.2. Respiratory Effects

Zebrafish studies have demonstrated respiratory impairments following e-cigarette exposure, including changes in gill morphology and reduced oxygen uptake [116,125,126]. In humans, e-cigarette use has been associated with lung damage and an increased risk of respiratory symptoms [127,128,129,130,131].

### 8.3. Neurobehavioral Effects

Both Zebrafish and human studies have reported neurobehavioral effects following e-cigarette exposure. Zebrafish exposed to e-cigarette aerosols altered locomotor activity and anxiety-like behaviors [59,126,132,133,134,135], similar to human behavioral changes [136,137].

### 8.4. Cellular and Molecular Mechanisms

Zebrafish studies have identified increased oxidative stress markers and evidence of cellular damage [138,139]. Similarly, human studies have indicated oxidative stress and inflammatory responses in e-cigarette users [140,141,142,143].

While zebrafish studies offer valuable insights into the potential toxicity and developmental effects of e-cigarettes, it is essential to acknowledge that Zebrafish are not perfect proxies for human biology. Variations in metabolism, organ structure, and overall physiology between species may lead to different responses to e-cigarette exposure. Furthermore, human e-cigarette usage is more complex, involving various devices, e-liquid formulations, and user behaviors, making direct comparisons between zebrafish and human findings challenging.

Despite these limitations, zebrafish studies serve as valuable preliminary models for understanding the potential effects of e-cigarette exposure. However, human studies remain essential for providing comprehensive and directly applicable data to assess the health impacts of e-cigarettes on human populations. As e-cigarette research progresses, further integrated studies involving Zebrafish, other model organisms, and human clinical research will contribute to a more comprehensive understanding of the potential risks associated with e-cigarette use.

## 9. Pushing the Boundaries of E-Cigarette Research with Zebrafish Models

As the use of zebrafish models in e-cigarette research continues to evolve, there is a growing need for innovative experimental approaches to deepen our understanding of the health implications of e-cigarette exposure. Here are several proposed methodologies from zebrafish studies that can be applied to advance e-cigarette research:

### 9.1. Organ-on-a-Chip Technology

Recent insights into dynamic cellular environments and intercellular communication have emphasized the importance of authentic organ function representation. Organ-on-a-chip technology, which provides predictive human tissue models and advanced tissue assembly techniques, holds promise for bridging gaps in drug screening [64]. Targeted toxicological assessments relevant to e-cigarettes can be conducted by integrating zebrafish embryos with organ-on-a-chip technology. This approach mimics human organs, offering insights into the cellular-level effects of e-cigarette exposure. This technology has been successfully employed to evaluate the respiratory hazards associated with fine particulate matter (PM2.5) by replicating the intricate anatomical structure of the lung [144]. Similar approaches can be used to investigate the effects of electronic cigarettes on Zebrafish by cultivating zebrafish cells on microfluidic chips and exposing them to e-cigarette aerosols or vapor, replicating real-life inhalation circumstances.

### 9.2. Single-Cell RNA Sequencing

The cutting-edge technique of single-cell RNA sequencing (scRNA-seq) has emerged as a powerful method for deciphering the diversity and complexity of RNA transcripts within individual cells. Leveraging this technique on Zebrafish exposed to e-cigarettes can reveal gene expression changes at the cellular level, shedding light on molecular responses to e-cigarette aerosols [66,145]. For example, recent investigations have used scRNA-seq to explore the influence of nicotine on human embryonic cardiogenesis, uncovering notable downregulation of specific cell types and key genes pivotal for cardiac development in response to nicotine exposure [146].

### 9.3. Gene Editing Technologies (CRISPR-Cas9)

Genome editing technologies like CRISPR-Cas9 enable the precise manipulation of genes in zebrafish, aiding in understanding e-cigarette toxicity mechanisms [147,148,149]. CRISPR-Cas9 is known for its swiftness, cost-effectiveness, precision, and efficiency compared to other genome editing techniques, making it a valuable tool for researchers.

### 9.4. Real-Time Imaging Techniques

Investigating cellular functions, developmental mechanisms, and in vivo processes often requires complex bio-imaging. Real-time imaging techniques like confocal or light-sheet microscopy capture dynamic processes in zebrafish embryos exposed to e-cigarette aerosols, providing visual insights into developmental changes [150]. This technology has been widely used in studies focused on electronic cigarette effects on physiological processes and organogenesis [107].

### 9.5. High-Resolution Mass Spectrometry

Mass spectrometry imaging is employed to construct molecular atlases for zebrafish larvae, facilitating the examination of specific molecules in different tissues. This technology can identify e-cigarette aerosol constituents and toxicants within zebrafish, aiding in the analysis of potential chemical exposures [151,152]. Integrating this technology with complementary assays offers a powerful approach to examining complex compounds in electronic cigarettes and understanding their health effects [153].

### 9.6. Longitudinal Studies

Longitudinal studies tracking zebrafish development over time after e-cigarette exposure provide valuable information about potential long-term health effects. This approach is crucial for comprehensively assessing the impacts of e-cigarette exposure and refining regulatory decisions and public health protocols. Given the limited number of longitudinal studies on the long-term effects of e-cigarette usage on human health and fetal development, such scientific endeavors hold paramount importance [154].

### 9.7. Multi-Omics Approaches and Behavioral Profiling

Integrating data from various biological layers, such as the genome, proteome, transcriptome, metabolome, lipidome, and microbiome/metagenome, enhances the power of zebrafish models to provide a complete understanding of e-cigarette effects [155,156]. Multi-omics approaches and comprehensive behavioral profiling reveal the intricate molecular dynamics underlying health and disease. These methodologies have been successfully employed in studies focused on understanding the molecular consequences of exposure to heated tobacco products [157].

By incorporating these innovative approaches into e-cigarette research using zebrafish models, researchers can uncover complex mechanisms and health implications associated with e-cigarette exposure. Integrating zebrafish findings with other model systems and human data is crucial for a comprehensive evaluation of e-cigarette toxicity. This integration strengthens the scientific basis for policy-making and public health protection by corroborating toxicological mechanisms through epidemiological data, biomarker studies, and risk assessments. 

## 10. Challenges and Limitations in Utilizing Zebrafish as a Model for E-Cigarette Toxicity Research

While zebrafish offer several advantages as a model organism for studying the toxicity of e-cigarettes, they also present notable challenges and limitations that must be considered. These limitations encompass various aspects, ranging from anatomical and physiological differences to experimental methodologies.

### 10.1. Variability in Anatomy, Physiology, and Metabolism

Zebrafish and humans differ significantly in terms of anatomy, physiology, and metabolism. These inherent disparities raise concerns about the direct translation of findings from zebrafish studies to human health. Zebrafish possess a simpler circulatory system, with two-chambered hearts, in contrast to the four-chambered hearts of humans. Their respiratory system also differs, as zebrafish primarily rely on gill respiration, unlike humans, who respire through the lungs. Additionally, zebrafish metabolize substances differently due to variations in enzymatic pathways. These distinctions may result in differing responses to toxic substances, limiting the generalizability of zebrafish findings to human populations.

### 10.2. Waterborne Exposure vs. Inhalation

A significant challenge in zebrafish studies is the mode of exposure to e-cigarette aerosols or constituents. Zebrafish are typically exposed to these substances through waterborne exposure, which does not accurately replicate the inhalation route of exposure in humans. Human smoking involves inhaling aerosols directly into the respiratory system, a process that significantly differs from zebrafish immersion in contaminated water. This discrepancy in exposure routes may lead to variations in the absorption, distribution, and metabolism of e-cigarette constituents, making it challenging to draw direct parallels between zebrafish outcomes and human health effects.

### 10.3. Dose Discrepancies

The doses administered in zebrafish experiments may not encompass the full spectrum of human exposures to e-cigarettes. Human e-cigarette users exhibit substantial variability in device types, e-liquid formulations, and consumption patterns. These diverse factors contribute to a wide range of exposure levels, which may not be accurately replicated in zebrafish experiments. Consequently, zebrafish findings may not fully capture the potential health risks associated with the breadth of human e-cigarette usage patterns.

### 10.4. Developmental Timing and Short Lifespan

Zebrafish are renowned for their rapid development and relatively short life cycle. While this characteristic allows for quick observations and experimental turnover, it raises concerns regarding the applicability of findings to the long-term effects of chronic e-cigarette use in humans. Many zebrafish studies focus on early developmental stages, potentially missing the protracted and cumulative health impacts that manifest over extended periods in humans. The short lifespan of Zebrafish further limits the assessment of chronic and age-related effects observed in human e-cigarette users.

### 10.5. Behavioral Assessments and Cognitive Functions

Behavioral assessments in zebrafish studies provide valuable insights into the effects of e-cigarette exposure; however, these assessments may not comprehensively reflect human behaviors and cognitive functions. Zebrafish behaviors are inherently simpler than those of mammals, making it challenging to extrapolate zebrafish behaviors directly to complex human behaviors. This discrepancy complicates the translation of zebrafish findings into comprehensive insights on e-cigarette toxicity in humans, particularly regarding intricate interactions among various e-cigarette constituents.

While zebrafish studies play a crucial role in the initial toxicological screening and mechanistic exploration of e-cigarette toxicity, they are not without limitations. These constraints underscore the importance of complementing zebrafish research with diverse model organisms and human clinical studies. A comprehensive approach combining insights from multiple sources will provide a more thorough understanding of the potential health risks associated with e-cigarette use, ultimately aiding in formulating informed public health policies and interventions.

## 11. Conclusions

In conclusion, zebrafish research has been indispensable in understanding cellular and developmental responses associated with e-cigarette exposure. Findings consistently demonstrate that, despite some differences between zebrafish and humans or mammals, this model offers a unique opportunity to anticipate health risks associated with e-cigarette consumption in vulnerable populations, such as pregnant women and fetuses. Thus, prompt and legal interventions can be developed to safeguard the well-being of those most susceptible to the potential risks posed by e-cigarettes. As we continue to navigate the complex landscape of e-cigarette use, the contributions of zebrafish research remain invaluable in safeguarding the health and well-being of individuals and communities worldwide, proving its relevance to human health.

## Figures and Tables

**Table 3:** Main aspects of the relevance of zebrafish cardiovascular development to human health.

Aspect	Relevance to Human Health
Conservation of genes and pathways	Shared genes and pathways with humans provide insights into heart development and diseases [70].
Modeling cardiovascular diseases	Zebrafish models mimic human heart conditions, aiding disease mechanism exploration [71].
Drug discovery and toxicity testing	Transparent embryos enable drug testing and safety assessment, expediting drug development [54,55].
Heart regeneration	Studying zebrafish heart regeneration informs human cardiac tissue repair research [72,73,74,75].
Functional analysis of disease-associated genes	Genetic manipulation studies reveal the effects of disease genes, aiding understanding of human disorders [69,75].
Personalized medicine and therapies	Zebrafish models allow testing patient-specific genetic variants, guiding personalized treatments [76,77,78].

## Data Availability

No data were used for the research described in the article.
